# Effects of gene therapy on muscle 18S rRNA expression in mouse model of ALS

**DOI:** 10.1186/1756-0500-3-275

**Published:** 2010-11-02

**Authors:** María Moreno-Igoa, Raquel Manzano, Sara Oliván, Ana C Calvo, Janne M Toivonen, Rosario Osta

**Affiliations:** 1 LAGENBIO-I3A, Veterinary Faculty, Aragon Institute of Health Sciences (IACS), Universidad de Zaragoza, Miguel Servet 177, 50013 Zaragoza, Spain

## Abstract

**Background:**

The efficiency of gene therapy experiments is frequently evaluated by measuring the impact of the treatment on the expression of genes of interest by quantitative real time PCR (qRT-PCR) and by normalizing these values to those of housekeeping (HK) genes constitutively expressed throughout the experiment. The objective of this work was to study the effects of muscle gene therapy on the expression of 18 S ribosomal RNA (*Rn18S*), a commonly used HK gene.

**Findings:**

Mouse model of motor neuron disease (SOD1-G93A) was injected intramuscularly with Brain-derived neurotrophic factor (BDNF-TTC) encoding or control naked DNA plasmids. qRT-PCR expression analysis was performed for BDNF and HK genes *Rn18 S*, glyceraldehyde-3-phosphate dehydrogenase (*Gapdh*) and β-actin (*Actb*). We report that elevated *BDNF *expression in the injected muscle was accompanied with increased *Rn18 S *expression, whereas *Gapdh *and *Actb *were not affected. Increased "ribosomal output" upon BDNF stimulation was supported by increased steady-state levels of ribosomal protein mRNAs.

**Conclusions:**

Ribosomal RNA transcription may be directly stimulated by administration of trophic factors. Caution should be taken in using *Rn18 S *as a HK gene in experiments where muscle metabolism is likely to be altered by therapeutic intervention.

## Background

Quantitative Real Time PCR (qRT-PCR) is an increasingly popular method for the quantitative analysis of gene expression. Despite its high sensitivity, accuracy and wide dynamic range that favour qRT-PCR in gene expression studies, some factors exist that must be taken into account as a possible source of error [1]. A critical element in experimental design is the strategy to quantify the input template cDNA in the sample. Appropriate choice of internal references has been previously shown to be crucial for correct interpretation of expression data [1,2] and bioinformatic approaches have been developed to increase the accuracy of normalization [3-5]. Although numerous reference genes are currently used for normalization purposes, the most commonly used are still 18 S ribosomal RNA *(Rn18S)*, β-actin *(Actb) *and glyceraldehyde-3-phosphate dehydrogenase *(Gapdh*) due to their ubiquitous and relatively high expression levels [6]. *Actb *and *Gapdh *are mRNA-encoding housekeeping genes (HKs), and have been claimed to be either suitable or unsuitable as reference genes depending on tissue or experimental conditions used [6-10]. On the other hand, *Rn18 S *encodes ribosomal RNA (rRNA). Although rRNAs are highly abundant and, therefore, untypical RNA-species in the cell, *Rn18 S *has been described to maintain stability under some conditions that may result in altered housekeeping mRNA levels [7]. *Rn18 S *has been regarded as appropriate endogenous control in experiments including cell culture [11,12] and tissue biopsies [13].

In metabolically active cells rRNA genes are transcribed efficiently to keep up with high demand for protein synthesis machinery. Traditional northern RNA quantification has favoured *Rn18 S *because of its conveniently high expression level which can dramatically reduce the time required for the autoradiographic detection. However, when qRT-PCR with relative quantification is used, high abundance of *Rn18 S *compared with target mRNA transcript becomes a hindrance as it complicates accurate subtraction of the baseline value in real-time qRT-PCR data analysis [5]. As opposed to mRNA genes (such as *Actb *and *Gapdh*) that are transcribed by RNA polymerase II (Pol II), rRNA transcription is dependent on RNA polymerase I (Pol I) devoted exclusively to this task. Pol I activity is a crucial determinant for production of ribosomes needed for growth and cell proliferation [14,15]. Potential differences between regulatory networks modifying transcriptional activity of Pol I and Pol II is a major criticism for using rRNA genes for normalization. Availability of extracellular stimulatory factors (such as nutrition or growth factors), intracellular conditions (such as mutations), and drugs may alter mRNA and rRNA pools in dissimilar manner, or even to opposite directions [16,17]. Indeed, the hallmark of cancer is augmented rRNA transcription [18] and Rn18 S normalization has been shown to be potentially confounding in gene expression analysis of rat mammary tumors [19]. Pol I is a known target for growth-promoting signals such as epidermal growth factor [15] and insulin-like growth factor 1 [20]. This may well influence rRNA expression levels in cells where exogenous genes have been introduced to provide gene therapy, especially when introduced molecule stimulates anabolic pathways of the target cells.

Amyotrophic lateral sclerosis (ALS) is a devastating adult-onset motor neuron disease characterized by a progressive degeneration of motor neurons, skeletal muscle atrophy, paralysis and death. A well described mouse model of ALS, an overexpresser of human superoxide dismutase 1 (SOD1) carrying glycine to alanine substitution at residue 93 (SOD1-G93A) [21], recapitulates many features observed in human patients. Our previous work has established that the symptoms of the disease in this model can be alleviated with intramuscular injection of either recombinant proteins or "naked DNA" plasmids encoding neurotrophic factors, such as Glial cell-derived neurotrophic factor (GDNF) [22] or Brain-derived neurotrophic factor (BDNF)[23], coupled with atoxic C-terminal fragment of tetanus toxin (TTC) to enhance retrograde transport from muscle to neurons [24]. Besides delaying a functional decline and lethality of SOD1-G93A mice, these therapies activate Akt kinase by increasing PI3K-dependent signalling that promotes growth and survival [22].

The aim of the present study was to evaluate the effect of an exogenous BDNF-TTC fusion construct expression *in vivo *on the levels of *Actb*, *Gapdh *and *Rn18 S *in transfected tissue and, therefore, validation of these HK genes as an endogenous reference in such gene therapy studies.

## Results and discussion

Briefly, BDNF-TTC-encoding (pcDNA3.1-pCMV-BDNF-TTC) or non-coding control (pcDNA3.1-pCMV) naked DNA plasmids were each injected intramuscularly into the quadriceps of ten SOD1G93A transgenic mice at 60 days of age (asymptomatic stage). Each muscle was injected with total 100 μg of plasmid in physiological saline, in two 50 μL injections. Ten days or fifty days after injections (at ages of 70 days and 110 days, respectively) the animals were anaesthetized with pentobarbital (50 mg/kg) and sacrificed by cervical dislocation. Quadriceps muscles were snap-frozen in liquid nitrogen and stored at -70°C. All experimental procedures were approved by Ethics Committee of our institution and followed the international guidelines for the use of laboratory animals. For gene expression analysis, total RNA extracted from frozen muscle tissue of each animal was DNase treated and retrotranscribed, and the cDNA was used for the expression analysis of plasmid-derived BDNF (BDNF-TTC) as well as that of HK genes *Rn18S*, *Gapdh *and *Actb *(see full details in additional file 1). Relative expression levels of BDNF and *Rn18 S *were normalized with the geometric mean of those of *Actb *and *Gapdh *[5]. For the expression stability analysis of *Actb *and *Gapdh*, the transcripts were normalized with each other. Relative gene expression compared with control plasmid-injected animals was determined using the 2^-ΔΔCT ^method [25]. Student's t-test was used and statistical differences were considered significant at P ≤ 0.05 (Statistica 5.0).

**Figure 1:**
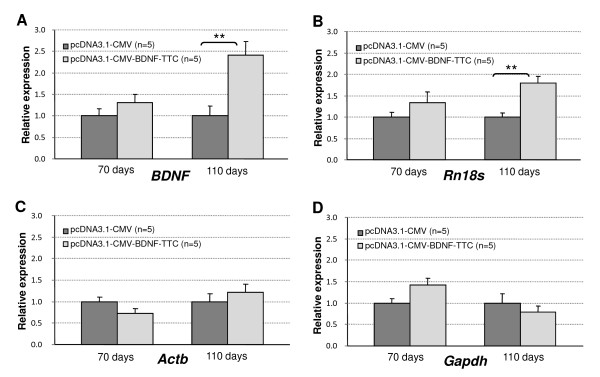
**Up-regulation of Rn18 S upon BDNF-treatment**. Gene expression analysis in muscle tissue of SOD1-G93A animals intramuscularly injected with pcDNA3.1-CMV control plasmid (dark grey bars) or pcDNA3.1-CMV-BDNF-TTC plasmid (light grey bars) at ten days (age 70 days) or fifty days (age 110 days) post-injection. A) *BDNF *expression is unchanged (P = 0.258) at age of 70 days but shows 2.4-fold increase (P = 0.007) in therapeutically treated animals at age of 110 days. B) *Rn18 S *expression is not altered at age of 70 days (P = 0.298) but is 1.8-fold higher (P = 0.009) at age of 110 days. C) *Actb *and D) *Gapdh *expression is unaltered in all conditions (P > 0.05). Error bars indicate standard error of mean. Symbol ** denotes statistical significance level P ≤ 0.01.

At day 10 post-injection there were no significant differences in the expression of BDNF between animals treated with pcDNA3.1-pCMV-BDNF-TTC plasmid and those treated with pcDNA3.1-pCMV control vector (Figure 1A, left). Accordingly, we did not find significant differences in the *Rn18 S *gene expression between these groups (Figure 1B, left). By contrast, 50 days post-injection the expression of BDNF was 2.4-fold higher in pcDNA3.1-pCMV-BDNF-TTC treated animals (p < 0.01), which correlated with a 1.8-fold increase in the transcripts of *Rn18 S *gene (p < 0.01) (Figures 1A and 1B, right). On the other hand, neither *Actb *nor *Gapdh *showed significant differences between control and pcDNA3.1-pCMV-BDNF-TTC treated animal groups at 70 or 110 days of age (Figures 1C and 1D). These observations are consistent with a previously described role of BDNF in upregulating Pol II-dependent (mRNA) components of the translation machinery [26], and possibly indicate also increased Pol I transcriptional activity in the treated muscle in response to BDNF. Although the observed 1.8-fold upregulation of *Rn18 S *upon BDNF-TTC treatment may seem small compared with changes often reported to mRNA genes, this degree of Pol I transcriptional activation has been described in growth factor-stimulated cells [15,20]. Since transcription of rRNA genes utilizes as much as 40-50% of the transcriptional capacity in mammalian cells [27], even two-fold relative increase in *Rn18 S *transcripts is significant in absolute quantities.

**Figure 2:**
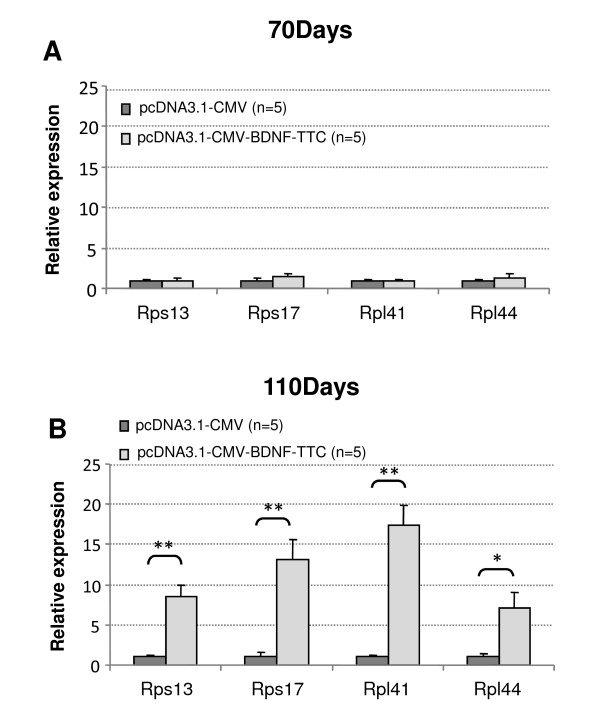
**Up-regulation of ribosomal protein mRNAs upon BDNF-treatment**. Gene expression analysis was carried out as in Figure 1. pcDNA3.1-CMV control plasmid -treated muscles are shown as dark grey bars and pcDNA3.1-CMV-BDNF-TTC plasmid treated muscles as light grey bars. A) Transcripts for ribosomal proteins *Rps13*, *Rps17*, *Rpl41 *and *Rpl44 *were unaffected (P = 0.854, P = 0.472, P = 0.735 and P = 0.522, respectively) by BDNF-treatment at age of 70 days. B) Transcripts for ribosomal proteins were increased by BDNF-treatment at age of 110 days: *Rps13 *(8.6 fold, P < 0.01), *Rps17 *(13.1-fold, P < 0.01), *Rpl41 *(17.4-fold, P < 0.01) and *Rpl44 *(7.1-fold, P < 0.05). Error bars indicate standard error of mean. Symbols ** and * denote statistical significance level P ≤ 0.01 and P ≤ 0.05, respectively.

To further investigate if the observed *Rn18 S *increase upon BDNF-treatment reflects a general increase in ribosomal output we compared the expression of four "muscle enriched" [28] ribosomal protein mRNAs (two from small 40 S subunit and two from large 60 S subunit) using the same cDNA samples. The steady state mRNA levels of *Rps13*, *Rps17*, *Rpl41 *and *Rpl44 *(also known as *Rpl36a*) showed 7-17-fold increase in BDNF-treated compared with control plasmid treated muscles (Figure 2). Wheather this increase reflects increased Pol II transcription, increased mRNA stability, or both, remains unknown. However, these results are consistent with general induction of the translation machinery by BDNF [26].

Increasing evidence indicates involvement of rRNA suppression during pathogenesis of motor neuron disease. rRNA synthesis takes place in the nucleoli, which are the sites of ribosome biogenesis in the eukaryotic cells, and perturbation of nucleolar function leads to neurodegeneration in mice [29]. Haploinsufficiency of angiogenin (ANG) has been linked to the pathogenesis of ALS, and ANG is known to stimulate rRNA transcription by direct transcriptional regulation as well as to activate synthesis of ribosomal proteins by stimulation of Akt/PI3K survival pathway [30]. We propose that the increase in the *Rn18 S *transcript levels reflects a stimulus of the translation machinery occurring in the muscles and/or neuromuscular junctions of the BDNF-TTC treated SOD1-G93A animals. BDNF treatment can activate Akt/PI3K [22] and ERK1/2 [31] signalling pathways, which are, respectively, required for stimulation of Pol I-dependent rRNA transcription upon IGF-1 treament [20] and EGF treament [15]. BDNF has been recently shown to potentiate *in vivo *muscle regeneration after toxin-induced damage, and this activity may derive from its stimulatory effect on function of muscle stem cells, satellite cells [32]. Although we did not specifically study satellite cells here, it seems possible that cell cycle activation in this normally quiescent supply of muscle progenitors may at least partially contribute to the observed induction in *Rn18S*. Indeed, transcription of both rRNA [33] and ribosomal protein mRNAs [34] is increased in proliferating myoblasts compared with mature myofibers. Our results are also in agreement with those reported earlier [35] where considerable variation in *Rn18 S *expression in skeletal muscle was observed in response to intense exercise which is known to activate metabolism and differentiation status of myogenic and mature muscular cells.

Discrepancies exist about the utility of *Rn18 S *in normalization in other types of cells. In activated lymphocytes *Rn18 S *transcript levels remain relatively stable compared to unstimulated ones [36]. Similarly, constitutive expression of *Rn18 S *was described in myeloid leukaemia cell lines when stimulated to differentiate although, upon stimulation of apoptosis using the same cell lines, *Rn18 S *was found to be unreliable reference gene [37]. Thus, it seems that the usefulness of *Rn18 S *for normalization purposes depends on both cell type and experimental intervention that tissue is subjected to. However, as discussed earlier, Pol I and Pol II transcription are subjects to differential regulation, which is the primary concern in using rRNAs for mRNA normalization. Data presented here and by others [35] indicate instability of *Rn18 S *under two conditions that stimulate muscle cell activity, namely gene therapy and exercise. Therefore, qRT-PCR data normalization using *Rn18 S *in muscle tissue under regenerative treatment or exercise is clearly not recommended.

Molecules that provide trophic support to the atrophic muscle are under vigorous investigation since they are predicted to be beneficial in patients suffering from muscular or neuromuscular diseases, and may improve the recovery from traumatic damage [38,39]. Therefore, poor performance of *Rn18 S *as a HK gene needs a special recognition in the regenerative therapy field, and the same may well apply to the mRNAs encoding components of the translation machinery. On the positive note, the results presented here potentially reveal the utility of increased *Rn18 S *transcript levels as a surrogate marker to measure the effectiveness of therapeutic interventions in muscular and neuromuscular diseases.

## Competing interests

The authors declare that they have no competing interests.

## Authors' contributions

MMI and RM carried out the mouse gene therapy work and tissue extraction and analyzed the gene expression. SO and ACC performed the statistical analysis and participated in the design of the work. JMT and RM wrote the manuscript. RO designed and initiated the project and supervised the work. All authors have read and approved the manuscript.

## Supplementary Material

Additional file 1 **Methodological details**. A detailed description of animal housing, RNA extraction, retrotranscription and quantitative real time PCR analysis. Click here for file
