# Supplementary material for: Effects of gene therapy on muscle 18S rRNA expression in mouse model of ALS
Source: BMC Res Notes. 2010 Nov 2;3:275. doi: 10.1186/1756-0500-3-275 (PMC2987871; doi:10.1186/1756-0500-3-275)
Supplement: Additional file 1 — Methodological details. A detailed description of animal housing, RNA extraction, retrotranscription and quantitative real time PCR analysis. [file 1756-0500-3-275-S1.DOC]

# Additional files

## Additional file 1

**Animal housing:** The transgenic mice B6SJL-Tg(SOD1-G93A)1Gur/J expressing G93A mutant form of human *SOD1* [12] were purchased from The Jackson Laboratory (Bar Harbor, ME, USA) and were housed under a 12h light: 12h dark cycle in 21–23°C with relative humidity of 55%. Food and water were available *ad libitum*. Transgenic colony was maintained by breeding hemizygous SOD1G93A males with wild type female littermates. The genotypes were determined from tail samples as described in The Jackson Laboratory protocol.

**RNA extraction and** r**etrotranscription**: Frozen muscle tissue was pulverized in a cold mortar using liquid nitrogen and RNA was extracted using TRIzol Reagent (Invitrogen S.A, Prat de Llobregat, Barcelona, Spain) according to manufacturers instructions. To eliminate genomic DNA, samples were treated with Ambion Turbo DNA-free kit (Applied Biosystems Inc, Foster City, CA, USA). Retrotranscription was carried out using SuperScriptTM First-Strand Synthesis System kit (InvitrogenS.A, Prat de Llobregat, Barcelona, Spain) using random hexamers with 1 μg of template RNA in a final volume of 20 μL.

**Quantitative real time PCR**: Expression of plasmid-derived BDNF (BDNF-TTC) in injected muscles was subsequently quantified by qRT-PCR in 10 μL reactions containing 150 nM of each primer (BDNF-forward, 5’- GGTCACAGCGGCAGATAAAAAGAC-3’ and BDNF-reverse, 5’- TTGGGTAGTTCGGCATTGCGAG- 3’), 5 μL of 2X SYBR® Green PCR Master Mix (Applied Biosystems Inc, Foster City, CA, USA) and 1 μL of 1:10 diluted cDNA. The thermal cycler parameters were: 95ºC for 2 min followed by 40 cycles of 94ºC for 30 s, 57ºC for 30 s and 72ºC 40 s. Quantification of the *Rn18S* expression was carried out in 10μL reactions containing 1X TaqMan® Universal PCR Master Mix (Applied Biosystems Inc, Foster City, CA, USA) with 1X of the supplied primer and TaqMan® MGB probe mix, and 1μL of 1:10 diluted cDNA per reaction. The thermal cycler parameters were: 95ºC for 10 min followed by 40 cycles of 95ºC for 15 s and 60ºC for 1 minute. Primer/probe mixtures for *Gapdh* and *Actb*, as well as for ribosomal proteins *Rps13*, *Rps17*, *Rpl41* and *Rpl44* (also known as *Rpl36a*) were designed and supplied by Applied Biosystems (Applied Biosystems Inc, Foster City, CA, USA) and reaction conditions were identical to those of *Rn18S*. Each reaction was performed in triplicate using an ABI Prism 7000 Sequence Detection System (Applied Biosystems Inc, Foster City, CA, USA) and reaction efficiencies of the primer/probe sets were close to 100%.
